# High-dose rifampicin in tuberculosis: Experiences from a Dutch tuberculosis centre

**DOI:** 10.1371/journal.pone.0213718

**Published:** 2019-03-14

**Authors:** Charlotte Seijger, Wouter Hoefsloot, Inge Bergsma-de Guchteneire, Lindsey te Brake, Jakko van Ingen, Saskia Kuipers, Reinout van Crevel, Rob Aarnoutse, Martin Boeree, Cecile Magis-Escurra

**Affiliations:** 1 Department of Pulmonary Diseases, Radboud University Medical Center-Dekkerswald, Nijmegen, The Netherlands; 2 Department of Pharmacy, Radboud University Medical Center, Nijmegen, The Netherlands; 3 Department of Medical Microbiology, Radboud University Medical Center, Nijmegen, The Netherlands; 4 Department of Internal Medicine, Radboud University Medical Center, Nijmegen, The Netherlands; Hospital Universitari de Bellvitge, SPAIN

## Abstract

**Background:**

Recent evidence suggests that higher rifampicin doses may improve tuberculosis (TB) treatment outcome.

**Methods:**

In this observational cohort study we evaluated all TB patients who were treated with high-dose rifampicin (> 10 mg/kg daily) in our reference centre, from January 2008 to May 2018. Indications, achieved plasma rifampicin exposures, safety and tolerability were evaluated.

**Results:**

Eighty-eight patients were included. The main indications were low plasma concentrations (64.7%) and severe illness (29.5%), including central nervous system TB. Adjusted rifampicin dosages ranged from 900 mg to a maximum of 2400 mg (corresponding to 32 mg/kg) per day. Patients with severe illness received high-dose rifampicin immediately, the others had a higher dosage guided by therapeutic drug monitoring. Four patients developed hepatotoxicity, of which two were proven due to isoniazid. Re-introduction of high-dose rifampicin was successful in all four. Eighty-seven patients tolerated high-dose rifampicin well throughout treatment. Only one patient required a dose reduction due to gastro-intestinal disturbance.

**Conclusion:**

High-dose rifampicin, used in specific groups of patients in our clinical setting, is safe and well-tolerated for the whole treatment duration. Measurement of drug exposures could be used as a tool/guide to increase rifampicin dosage if a reduced medication absorption or a poor treatment outcome is suspected. We suggest to administer high-dose rifampicin to patients with severe manifestations of TB or low rifampicin exposure to improve treatment outcome.

## Introduction

Rifampicin plays a key role in tuberculosis (TB) treatment regimens, due to its bactericidal and sterilizing capacity. It was introduced in the early 1970s at a dose of 10 mg/kg (with a maximum of 600 mg) once daily, mainly because of financial considerations and fear of toxicity[1]. More recently, several studies have suggested that the standard dose of 10 mg/kg rifampicin is suboptimal and at the lower end of the dose-response curve[1, 2]. This standard suboptimal dose of rifampicin may contribute to the emergence of new multi-drug resistant TB cases[3–7].

Optimisation of the rifampicin dosage has potential to improve treatment outcome and may shorten therapy duration[2, 8]. Several phase II randomized clinical trials (RCTs) have shown that increasing the rifampicin dose results in a non-linear increase in plasma concentrations[2, 9, 10]. A daily dosage of 35 mg/kg was found to be safe, well-tolerated, and resulted in a greater decline in bacterial load and reduced time to culture conversion[2, 10, 11]. In TB meningitis, an increased intravenous dosage of 13 mg/kg rifampicin resulted in a 50% reduced mortality in a phase II RCT in Indonesia [12]. An oral dose of 15 mg/kg rifampicin showed no benefit in a phase III RCT in Vietnam [12, 13], but in a recent phase II RCT from Indonesia, 30 mg /kg rifampicin orally was associated with decreased mortality [14].

Despite all these findings, the use of high-dose rifampicin (> 10 mg/kg) is not yet included in international TB treatment guidelines. The WHO appears to be awaiting additional evidence before adjusting its guidelines. To provide practical evidence for the use of high-dose rifampicin, we evaluated the indications, drug exposure, safety and tolerability of high-dose rifampicin in our TB expert centre during the past ten years.

## Methods

### Setting and subjects

All TB patients treated with a higher than standard (600 mg) daily dose of rifampicin in our TB reference centre from January 2008 until May 2018 were evaluated retrospectively. Patients are referred to our centre for treatment and isolation and normally stay admitted for several weeks (mean 6 weeks). After admission, follow up in the outpatient clinic is on a regular basis. Some patients left the country when no longer infectious to others, and thus are lost to follow up for this study. All patients in our TB database were screened for the use of high-dose rifampicin (>600 mg per day). Demographics, laboratory and pharmacokinetic results were recorded from patients’ electronically medical files. Patients were always informed about their indication for a higher than standard dose and possible adverse effects, unless they suffered from a state of decreased awareness or unconsciousness.

According to the ethics committee from the Radboud University Medical Centre, this study did not needed approval as this study did not fall within the remit of the Medical Research Involving Human Subjects Act (WMO). Patients who take part in investigations for clinical use, like in this study, automatically agree with the use of their data (anonymized) for research. If patients do not wish that their data will be used for clinical research they have to actively disagree. This study evaluated data which was primary obtained for clinical use and therefore it was not necessary to obtain informed consent. Although, the study has been reviewed by the ethics committee on the basis of the Dutch Code of conduct for health research, the Dutch Code of conduct for responsible use, the Dutch Personal Data Protection Act and the Medical Treatment Agreement Act. And a positive judgment on the study was passed by them.

All data were anonymized by the lead author.

### Therapeutic drug monitoring

Therapeutic drug monitoring (TDM) at our TB reference centre was used when abnormal rifampicin concentrations were suspected[15]: in case of relapse TB, delayed culture conversion (culture positive beyond two months of treatment), delayed clinical response, HIV co-infection, Diabetes Mellitus, suspicion of gastrointestinal malabsorption, severe weight loss or cachexia (BMI < 18.5 kg/m^2^), history of alcohol or drug abuse, renal or hepatic failure, and important drug interactions.

For TDM, blood samples were taken at 2, 4 and 6h after observed intake of drugs in a fasted state after at least ten days of treatment (steady state)[16, 17]. Bio-analysis was performed with validated liquid chromatographic methods [18]. The limited sampling strategy enabled estimation of peak plasma (C_max_) concentrations and total exposure (area under the plasma concentration versus time curve up to 24 h, AUC_0-24_)[16, 17]. In limited cases and in patients on intravenous rifampicin, more intensive PK sampling took place and PK measures were calculated using non-compartmental PK data-analysis using Phoenix Winnonlin (Pharsight Corp., Mountain View, CA, USA). Results for C_max_ and AUC_0-24_ were compared to average population measures, which are internationally used as reference values. The reasoning is that average exposures will result in a favorable treatment outcome in the majority of patients[15, 16]. The comparison between TDM and population PK measures is performed by the hospital pharmacist who provides a dosing advice based on the results, targeting for a C_max_ of ≥8 mg/L and an AUC_0-24_ ≥ 41 mg/L [16]. Actual adjustment of rifampicin dosage was decided by the physician based on TDM results, susceptibility of the causative mycobacteria and actual treatment response. After dose adjustment, tolerability, haematology and blood chemistry were evaluated on a regular basis. TDM after dose adjustment was only performed in case of suspected toxicity or under-dosing. The duration of treatment is always extended by the day of rifampicin dose adjustment as the new treatment start date.

### Statistics

Descriptive statistics were performed using IBM SPSS v. 23.0 software. Kolmogorov-Smirnov test was used to test for normality of distributions. Results were expressed as mean with a minimum to maximum, or as median with minimum to maximum in case of non-normality. C_max_ and AUC_0-24hs_ were presented as geometric mean and range, as PK parameters are not normally distributed in the general population.

## Results

### Patients and PK results

Data of eighty-eight patients on high-dose rifampicin were available for retrospective evaluation. High-dose rifampicin was used in patients with: severe illness including central nervous system (CNS) TB (n = 26); low plasma concentrations (n = 57); other reasons (n = 5). Patient characteristics and the indications for TDM are summarised in Table 1. The first group with severe illness consisted of twenty-six patients who were treated with high-dose rifampicin from treatment initiation, without establishing plasma concentrations first. Twenty patients with central nervous system (CNS) TB (15 TB meningitis, three cerebral tuberculomas, one myelitis and one with cerebral abscesses) and six with pulmonary TB (four extensive pulmonary cavities / consolidations, one with Acute Respiratory Distress Syndrome (ARDS) and one with sepsis and multi-organ failure). Five of the CNS TB patients received rifampicin intravenously.

**Table 1:** Characteristics of TB patients treated with high-dose rifampicin (n = 88).

Characteristics	N (%)^a^
**Demographics**	
Male	71 (80)
Age (years) [median]	45 (15–86)
Body weight (kg) [median]	60.5 (31–115)
Body Mass Index (kg/m^2^) [median]	20.5 (11.9–36.6)
**WHO region of patient origin**	
European Region	42 (47.7)
Eastern Mediterranean Region	21 (23.9)
African Region	14 (15.9)
South East Asia Region	6 (6.8)
Region of the Americas	5 (5.7)
**Type of TB**	
Pulmonary	47 (53.4)
Central nervous system	20 (22.7)
Other extrapulmonary	21 (23.9)
**Co-morbidities**^b^	
Diabetes Mellitus	17 (19.3)
Gastrointestinal tract anomalies	8 (9.1)
Renal failure	4 (4.5)
Liver cirrhosis	1 (1.1)
HIV co-infection	4 (4.5)
**Indications to perform TDM**^c^	N = 57
Relapse TB	12 (21.1)
Delayed sputum culture conversion	12 (21.1)
Delayed clinical response	14 (24.6)
HIV co-infection	4 (7)
Diabetes Mellitus	12 (21.1)
Gastrointestinal malabsorption^d^	8 (14)
Severe weight loss or BMI < 18.5 kg/m^2^	18 (31.6)
Alcohol abuse	12 (21.1)
History of drug abuse	7 (12.3)
Renal failure^e^	4 (7)
Liver cirrhosis	1 (1.8)
Rifampicin related drug interaction^f^	4 (7)

^a^ Results are expressed as *n* (%), or in case of age, body weight and BMI in median (minimum–maximum).

^b^ Other co-morbidities were respiratory, cardiovascular and auto-immune mediated diseases.

^c^ Multiple indications can be present in one patient. Sometimes TDM was indicated for other TB drugs than rifampicin (eg renal failure), but rifampicin was also measured.

^d^ Malabsorption due to abdominal tuberculosis localisation (n = 3), history of gastro-intestinal resection (n = 2), colitis ulcerosa (n = 1), presence of duodenal feeding tube (n = 1).

^e^ Three patients on haemodialysis and one patient with an estimated glomerular filtration rate of 50 ml/min.

^f^ Interaction with mesalazine (n = 2), antiretroviral medication (n = 1) and antiretroviral medication in combination with valproic acid, methadone and pregabalin (n = 1).

The second group consists of fifty-seven patients who had a dose adjustment of rifampicin after detecting low plasma concentrations. Table 2 shows in the colums group 1 and 2. The rows show the adjusted doses and the associated PK results (if available).

**Table 2:** Pharmacokinetic parameters of initial high dose rifampicin group in the severe illness group and dose adjustment group.

	Group 1	Group 2	
	(*n* = 26)	(*n* = 57)	
**Initial dose and pharmacokinetic parameters**		**Initial dose 450 mg**	**Initial dose 600 mg**
		*n* = 5	*n* = 52
Rifampicin dose (mg/kg)		11.4 (9.6–14.5)	17.7 (7.8–30.0)
C_max_*		2.8 (0.2–6.6)^5^	5.3 (1.5–13.6)^43^
AUC _0–24_*		26.5 (20.1–35.0)^2^	27.7 (8.8–65.7)^27^
**Adjusted dose and pharmacokinetic parameters**			
**Rifampicin 900mg**	*n* = 4	*n* = 3	*n =* 21
Rifampicin dose (mg/kg)	15.1 (13.1–17.3)	24.9 (21.4–29.0)	15.2 (7.8–20.9)
C_max_*	18.6^1^	15.6 (12.9–18.7)^3^	11.6 (6.5–22.1)^13^
AUC _0–24_*	105.0^1^	104.0 ^1^	58.4 (42.0–119.4)^8^
**Rifampicin 1200mg**	*n* = 18	*n =* 2	*n =* 28
Rifampicin dose (mg/kg)	18.0 (12.6–27.4)	22.9 (19.2–26.7)	18.6 (12.5–27.7)
C_max_*	19.3 (13.0–37.3)^8^	19.1^1^	16.8 (8.7–29.7)^13^
AUC _0–24_*	139.5 (103.0–250.0)^7^	79.0^1^	85.7 (47.0–150.0)^6^
**Rifampicin 1500mg**	*n* = 1		*n* = 1
Rifampicin dose (mg/kg)	29.4		30.0
**Rifampicin 1800mg**	*n* = 3		*n* = 1
Rifampicin dose (mg/kg)	30.2 (28.3–32.0)		28.6
C_max_			17.5
AUC _0–24_			117.0
**Rifampicin 2400mg**			*n* = 1
Rifampicin dose (mg/kg)			26.3
C_max_			37.8
AUC_0-24_			236

Group 1: initial high-dose rifampicin due to severe illness.

Group 2: proven low plasma concentrations on standard dose with the dose adjustments made guided by therapeutic drug monitoring.

Results of rifampicin dose per kg bodyweight are expressed as mean (minimum–maximum) and C_max_ and AUC_0-24_ are presented as geometric mean (minimum–maximum).

*Data available in number of patients.

AUC_0-24_, area under the 24-h concentration-time curve; C_max_, peak plasma concentration. Target C_max_ range: ≥8 mg/L and AUC_0-24_ of 41.1 mg/L, based on a publication in 2014 with population pharmacokinetics [16].

Five patients shifted to a higher rifampicin dosage because of persisting positive sputum culture at two months treatment (n = 2), presence of *rpoB* mutation which turned out to be located outside the ´hotspot´ region and with a normal phenotypic susceptibility (MIC < 0.25 mg/L) (n = 1), relapse TB with plasma concentrations only slightly above the target range (n = 1) and because of severe weight loss (n = 1).

### Safety and tolerability of high-dose rifampicin

High-dose rifampicin was well tolerated. All patients have finished a 6–12 months treatment course with an adjusted rifampicin dose. In terms of safety or tolerability, no difference was observed between 20 or 30 mg/kg. Drug induced liver injury developed in four patients (4.5%) (ALT range 243–899 U/L and AST range 242–1482 U/L (severity grade 3–4[19]). In two patients isoniazid-related hepatitis was proven with an isoniazid re-challenge. Re-introduction of high-dose rifampicin, after normalisation of the transaminases, was successful in all four patients. In six patients an elevated gamma GT (> 5 times upper limit of normal) was observed after dose adjustment. This remained either stable or decreased spontaneously during therapy. One patient with liver cirrhosis (Child Pugh class C; ascites, oesophageal varices and hypoalbuminemia) with bilirubin levels > 5 times the upper limit of normal before start of treatment, tolerated high-dose rifampicin (21 mg/kg) well. In the first 12 days of treatment his bilirubin levels further increased from 85 μmol/L to 106 μmol/L and then slowly decreased to 46 μmol/L over 4 months. A HIV-positive patient showed liver transaminases and gamma GT levels 5 times above the upper limit of normal at two occasions. The first episode was due to concurrent active hepatitis C. The second was related to anti-retroviral therapy. One patient died in the fifth month of treatment, due to the complications of a hydrocephalus. One frail 66-year-old patient needed a dose re-adjustment from 1200 mg to 900 mg (21 mg/kg), because of appetite loss. In 14 other elderly (> 65 years), high-dose treatment was tolerated well.

## Discussion

This is the first report about the use of high-dose rifampicin throughout TB treatment in daily practice, either immediately after hospitalization for severe TB, mostly TB meningitis (*n* = 26), or in patients with proven low rifampicin plasma concentrations (*n* = 57). In our experience, doses up to 32 mg/kg for the whole duration of therapy were tolerated well in 99% of our patients. In accordance to a recent study of Velasquez et al. [20] we did not observe more rifampicin-related adverse events in patients using high-dose rifampicin during our ten years clinical experience. The larger pill burden resulted in minor complaints in a few patients. C_max_ and AUC_0-24_ of high-dose rifampicin were roughly similar to earlier reported results, also showing a large inter-individual variance [2, 9, 10, 14].

In the seventies, the addition of rifampicin to TB treatment resulted in an important therapy shortening, although the optimal dose of rifampicin has never been established. The currently used standard dose (10 mg/kg or 600 mg daily in most populations) seems to increase the risk of relapses and the emergence of acquired drug resistance [5]. This is particularly relevant in isoniazid mono-resistant cases [21] and for the Beijing genotype of *M*. *tuberculosis* that may be much more tolerant to rifampicin than other genotypes [22, 23].

Phase I and II trials evaluating safety, tolerability, pharmacokinetics and bactericidal activity of rifampicin doses up to 35 mg/kg daily have already shown these doses to be safe, well tolerated, resulting in a faster reduction of the bacterial load and possibly lowering mortality rates in case of TB meningitis [2, 3, 10, 13, 14]. Currently, a study with dosages up to 50 mg/kg is in progress (clinicaltrials.gov NCT01392911). A recent ‘*in silico*’ simulation study showed greater early bactericidal activity for the 50 mg/kg dose[11]. After establishing the most optimal dose, a phase III trial may provide the necessary evidence for rifampicin’s ability to shorten TB treatment duration. Results from ongoing (clinicaltrials.gov NCT02581527) or planned phase III trials are not expected in the near future.

Based on the current available evidence together with our 10 years’ clinical experience, the rapid introduction of high-dose rifampicin in four high risk groups was suggested [24]): patients with TB meningitis, TB patients with HIV infection, Diabetes Mellitus and patients who are severely ill, as defined by the presence of extensive cavities or a low body mass index (<18 kg/m^2^). These patients have shown to have low plasma concentrations and worse treatment outcomes [13, 25–30]. The suggested use of high-dose rifampicin should first be restricted to TB expert centres and meticulously evaluated to collect all necessary information about efficacy, safety, tolerability and also outcome. To the best of our knowledge, two other European TB expert centres are also using high-dose rifampicin in risk groups (personal communication C. Lange, Borstel, Germany and O. Akkerman, Groningen, The Netherlands).

Our study has some limitations. Firstly, TDM was not performed in all patients and some results were missing. In case of a medical emergency like in TB meningitis, to our opinion no time should be lost reaching steady state before increasing the dose, as mortality is positively influenced in the first weeks after starting treatment [13]. Also, some TDM data were lost because of the introduction of a new electronical medical record system in 2013. Secondly, patients with a low body weight (< 50 kg) receiving a 600 mg dose were possibly not evaluated due to our inclusion criterion of a higher than 600 mg daily dose. Therefore, we may have missed patients who actually did receive a higher than standard dose of rifampicin. Thirdly, the retrospective design of the study implied that we could not perform a complete treatment outcome evaluation based only on our data. We considered introducing external information from our national TB registry retrospectively but concluded that evidence would not prove solid enough to evaluate treatment efficacy, as some of the patients returned to their home countries where obtaining follow up data is complex.

In conclusion, ten years of clinical experience have resulted in growing confidence in the safe use of high-dose rifampicin in TB treatment. Daily dosages of up to 32 mg/kg are tolerated well for the whole duration of treatment and showed an expected incidence of adverse reactions. Currently, in our daily practice, we recommend using high-dose rifampicin for high risk groups with severe illness and poor treatment outcomes, and for patients with (suspected) low TB drug concentrations without establishing TDM first. This may be a simple but effective strategy to improve TB treatment outcomes and safe lives.

## Supporting information

S1 File Database. (SAV)
